# The terrestrial evolution of metabolism and life – by the numbers

**DOI:** 10.1186/1742-4682-6-17

**Published:** 2009-08-27

**Authors:** Gregory C O'Kelly

**Affiliations:** 1 392 Pismo St., San Luis Obispo, CA 93401, USA

## Abstract

**Background:**

Allometric scaling relating body mass to metabolic rate by an exponent of the former (*Kleiber's Law*), commonly known as quarter-power scaling (QPS), is controversial for claims made on its behalf, especially that of its universality for all life. As originally formulated, Kleiber was based upon the study of heat; metabolic rate is quantified in watts (or calories per unit time). Techniques and technology for metabolic energy measurement have been refined but the math has not. QPS is susceptible to increasing deviations from theoretical predictions to data, suggesting that there is no single, universal exponent relevant to all of life. QPS's major proponents continue to fail to make good on hints of the power of the equation for understanding aging.

**Essentialist-deductivist view:**

If the equation includes a term for efficiency in the exponent, thereby ruling out thermogenesis as part of metabolism, its heuristic power is greatly amplified, and testable deductive inferences are generated. If metabolic rate is measured in watts and metabolic efficiency is a redox-coupling ratio, then the equation is essentially about the energy storage capacity of organic molecules. The equation is entirely about the essentials of all life: water, salt, organic molecules, and energy. The water and salt provide an electrochemical salt bridge for the transmission of energy into and through the organic components. The equation, when graphed, treats the organic structure as battery-like, and relates its recharge rate and electrical properties to its longevity.

**Conclusion:**

The equation models the longevity-extending effects of caloric restriction, and shows where those effects wane. It models the immortality of some types of cells, and supports the argument for the origin of life being at submarine volcanic vents and black smokers. It clarifies how early life had to change to survive drifting to the surface, and what drove mutations in its ascent. It does not deal with cause and effect; it deals with variables in the essentials of all life, and treats life as an epiphenomenon of those variables. The equation describes how battery discharge into the body can increase muscle mass, promote fitness, and extend life span, among other issues.

## Background

This paper is a prelude to a rigorous mathematical exploration of the physics of organic batteries and their significance for understanding many phenomena of life, which will be presented in another article.

The second law of thermodynamics states that in a closed system, one to which additional energy is not available, the tendency over time will be for that energy to be equally distributed throughout the system such that net energy transmission between elements of that system ceases. This is called heat death. Open systems, in contrast, are replenished with energy that allows them to avoid heat death, at the cost of increased entropy around them. The difference between closed and open systems is apparent when the battery is considered. A battery that is not rechargeable, called a primary cell, is a closed system. Its power output is dependent upon non-reversible chemical reactions that may be exhausted. In contrast, a battery that is rechargeable, called a secondary cell, is an open system. Its chemical processes are reversible with the capture of energy from outside it. Both types of systems can occur as chemical-energy-storage devices, in the form of structures of organic molecules with covalent bonds. To the extent that the bio-cell is like a battery, it is a secondary cell, an open system, some of whose covalent bonds are reversible. When energy is introduced to or expended by the cell, how the perturbation in energy storage is equilibrated throughout the system depends upon the electrical properties and histology of the biomass.

Thermodynamics has little relevance for open systems, aside from the equilibration of captured energy through those systems, especially if they function near equilibrium already. The capture and expenditure of chemical energy in the covalent bonds of biomass is the phenomenon of metabolism. The expenditure of that energy powers changes in the size, organization, and activity of the biomass. Metabolic rate becomes the recharge rate of that biomass, expressed in watts, a unit of power.

Kleiber's Law is a power law formulated by Max Kleiber in the 1930s to fit the data from his metabolic studies of animals [1]. Kleiber's Law, MR = αW^3/4^, describes the relationship of metabolic rate (MR) to the biomass W, raised to an exponent, and multiplied by a correctional constant, α. Max Kleiber analyzed bio-energy as heat energy, not chemical energy, for this is all that his instruments allowed. Originally the exponent of W was taken to be 2/3, according to the 1883 *surface hypothesis *of Max Rubner. This hypothesis related biomass surface area to its volume in a Euclidean approach to the estimation of heat retention and loss by the biomass, per unit of surface area. Kleiber later took the exponent to be 3/4, since his study of small mammals seemed to support this, though he could not say why. West, Brown, and Enquist (WBE) allegedly justified the larger exponent, theoretically, in the journal *Science*, arguing that the fineness of capillary branching allowed for greater efficiency of nutrient delivery to the cells of creatures large enough to have hearts [2]. However, they also claimed the equation applied to bacteria and to ecosystems.

The same study that found metabolic-rate-scaling for endotherms is far closer to the value predicted by Kleiber than that of ectotherms, on the basis of the study of oxygen and sugar consumption in 229 species [3], reported numerous deviations from Kleiberian predictions. The inclusion of thermogenesis in metabolism has clouded the relevance of the equation. White et al. note, after studying metabolic rates in 938 species: "there is no universal metabolic allometry and ... models that attempt to explain only quarter-power scaling of metabolic rate are unlikely to succeed" [4]. Da Silva et al. arrive at a similar conclusion about the lack of a universal exponent, though they claim it should still be possible to develop a unified theory for the allometric scaling of metabolism if its essentials are known [5].

As handled, Kleiber's Law also has no variable for the availability of energy, or for the efficiency of its capture and expenditure, both relevant to the metabolic recharge rate of all biomass. To amend this, the equation is altered to include a term, in the exponent, for metabolic efficiency (ME) of the biomass W. This term is a ratio of the efficiency of redox coupling between the biomass battery W, and the sources of chemical energy available to it, measured against loss to heat. ME is therefore a ratio of amperes of anabolism to amperes of catabolism. The recharge rate of the organic battery is influenced not just by the size of the mass, as Kleiber would have it, but also by the availability of energy to it, and by the ability of the organic battery to capture and expend this energy. These two are expressed as the denominator and numerator of ME. Biological organization is based upon and constrained by energetics.

**Figure 1:**
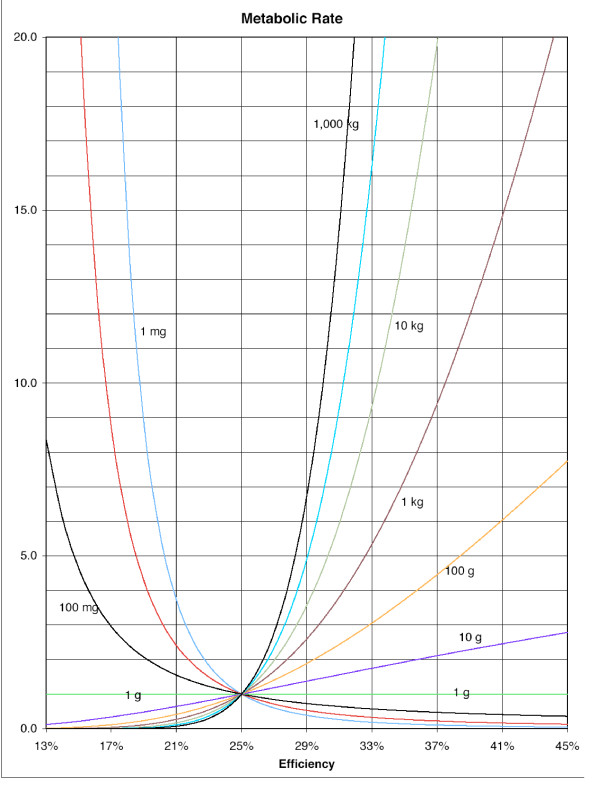
**Metabolic efficiency vs. metabolic recharge rate, with a different curve for each mass**. The curves shown are for the numbers highlighted in Additional file 1, and indicate how recharge rate changes for each mass as energy availability fluctuates, or as the ability of that mass to absorb energy changes. Both are expressed as values for metabolic efficiency, the X axis.

**Figure 2:**
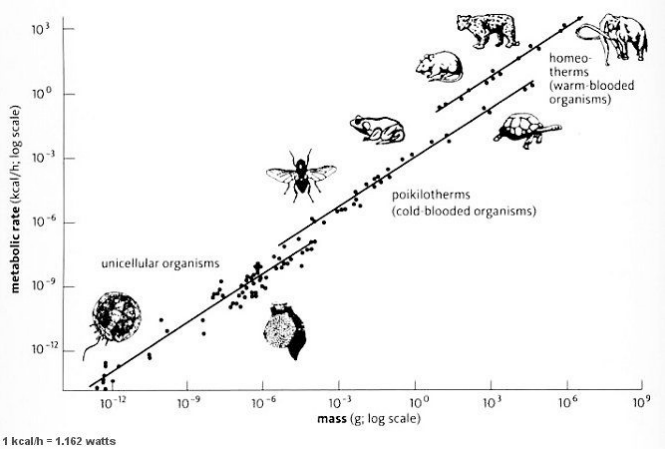
**The standard log-log graph of mass vs. metabolic rate, where each mass is a point on a line rather than a separate curve**. This graph is concordant with the archaic practice of including thermogenesis as part of metabolism, and the measuring of metabolism with the creature as close to equilibrium as possible, i.e., no food, no activity.

The sophisticated version of Kleiber's Law becomes MR = αW^(4ME-1)/4ME^. The ratio ME includes the effects of temperature on the electrochemical processes underlying redox coupling, but does not include the energy used to generate that temperature. When graphed with a different curve for each W (Fig. 1), values like those that appear in standard log-log graphs of mass vs MR occur when ME is between 100% (3/4) and 89% (2/3) (Fig. 2), something that never happens in biological or chemo-mechanical systems. The sophisticated version of Kleiber seems to remove the equation, as widely handled, from having any biological relevance whatsoever. This lack of relevance follows from misinterpretation of the role of thermogenesis in metabolism, which would account for the preposterously high exponents. Values for ME, realistically, are going to be much lower than 100%. At an ME of 20%, the exponent is -1/4. At around 33% the exponent is +1/4. At 25% ME, the exponent is 0. Fig. 1 reveals attractors at 25% ME and one gram mass. An interpretation of its curves follows. The highlighted numbers in Additional file 1 are those appearing as curves on Fig. 1, with a different curve for each value of W.

## An examination of graphs and table

Decrease in ME from increased energy availability (the denominator of the ratio) is the inefficiency of abundance (Ia). Increase in ME from decrease in energy availability is the efficiency of scarcity (Es). Ia and Es are passive changes due to circumstance. ΔME from Ia or Es results in changes in MR that are non-equilibrium deviations from an average MR. The immediacy and endurance of these perturbations pressures lagging, compensatory changes in the numerator of ME so that equilibrium is re-established. ΔME-driven fluctuations in MR are equilibrated by Ep (the efficiency of production), or by Id (the inefficiency of degradation). Both Ep and Id are due to changes in the numerator of ME. In the case of Ep, the organic battery functions as a secondary cell, where energy is captured as mass in reversible chemical reactions, e.g., as phosphorylation, glycogenesis, or the synthesis of NADH. In the case of Id the organic battery functions as a primary cell, where power is either expended or lost from catabolic reversal of anabolic chemical reactions, or its rate of capture is reduced. Equilibration pressure acts to change size, organization, and activity of W. For W less than one gram, increased Ia at less than 25% ME causes the MR of W to increase drastically, enchaining thermodynamic, equilibrating pressures to increase W through Ep, to lower MR. Absorption of energy as mass is met by MR drop. W, responsive to fluctuations in ME from Ia, is shaped by perturbations of MR that pressure for reversibility in W's structure. All Ws not able to absorb energy from Ia, as increased mass in the anabolic process of Ep, are destroyed by that energy, as Ia becomes Id.

At less than one gram, should increasing W from Ia reduce MR below average (given prevailing ME < 25%), equilibration pressure acting through Id reduces W and restores MR. Over time this reduction and its reverse will be selected for its reversibility, and will occur as division and growth. Sophisticated Kleiber represents replication/division and metabolism as inseparable, where replication is seen as reductions in biomass W due to the electrical properties of organic molecules, given ΔME and thermodynamic pressure for equilibrium.

The graph and table reveal key seams or attractors along the values one gram for W, and 25% for ME. These seams are not discontinuities. The lack of discontinuities from things as small as individual molecules, synthesized by a Miller/Urey lightning bolt for example, to large, single-celled organisms, further suggests that the growth of biomass to micelle and then to cell was continuous, and did not await a proto-cell wall of iron sulfide, or any other bioenergetic, structural arrangement with which some discontinuity that would mark the difference between life and non-life might occur. Instead, the origins of life appear as the result of aggregation of organic molecules. The boundary between life and non-life, it appears, is a matter of W's scale, where iteration of the redox dynamic is repeated at all scales.

This is seen in the life-like activity of calcium phosphate and carbonate particles once associated with what were called nano-bacteria [6]. Recent research has found a multitude of voltage differences within the cell that drive intracellular activity that can in no way be accounted for simply in terms of cell membrane voltages [7]. Sophisticated Kleiber models the evolution of form as not sudden or abrupt, but extremely gradual. Along these seams the equilibration of MR requires the least drastic changes in W for minor perturbations in MR.

Note that, at one gram, W's MR is the same for all MEs. Note also that at 25% ME the MRs for all Ws intersect. With ΔME occurring as passive changes in energy availability (Ia and Es), all Ws are met with changes in MR, except at one gram. The largest single-celled organisms, and the smallest mammal, are about one gram mass. It is no surprise that all life operates close to 25% ME, and that life's phases are frequently dictated by fluctuations to either side of 25% ME. Thermodynamics and unending fluctuations in ME drove evolution, from its beginning at submarine volcanic vents, where the secondary cell aspects of the bio-battery reigned in an energy-rich environment characterized both by ultraviolet radiation given off by a high-temperature mass in decompression cooling and magma formation, and by the richness of coulombs given off by the oxidation of hydrogen sulfide spewed into water to become sulfurous acid. This energy was absorbed as increased biomass, where the primary-cell aspects of the organic battery permitted the biomass to survive increased energy scarcity away from volcanic vents by allowing for reversible Id to counter Es.

The escape of W from high-energy environments awaited Ep's development then, facilitating not only mass increase, but also the organization of early W's constituents or organelles, so that Es from energy scarcity could be absorbed by Id. Mass increase, occurring as the formation of shells, the inclusion of iron in the structure (phytoplankton), or the aggregation of smaller w's into larger Ws (quorum sensing), at ME's over 25%, according to Fig. 1, results in increased MR for all Ws. At ME < 25%, however, increased W results in lower MRs. The rule is: passive ΔME over time (Ia and Es) triggers changes in the size and organization of W, by acting upon Id and Ep. These changes occur as proliferation, growth, and development of the organic battery, a storage device for coulombs. More importantly, as W drifted away from hydrothermal vents, changes in its structure allowed it to engulf particles upon which it could act catabolically, in the form of Id – acids acting upon covalent bonds, such that Id could be converted to Ia. This capacity supplemented the secondary/rechargeable cell aspects of early biomass with a primary battery aspect, at smaller scales, and ultimately became the activity of lysosomes and other aggregated, cellular organelles; and, at larger scales, became gastrulation. The delivery of these particles to the cells of sponges, corals, and tubeworms, at first dependent upon tides and currents, was later replaced by vascular systems in organic batteries with hearts.

## Changing scales

One cogent criticism leveled against WBE's handling of Kleiber, which claims to relate BMR to the W of the multi-cellular organism, is that BMR cannot possibly account for motor activity of W. Blood flow and primary cell-battery activity alone is not sufficient to power the great spikes in MR necessary for the sustained activity of large creatures. This is where FMR, the field MR of W that includes its activity, exceeds the BMR of its constituent parts (only at over 25% ME). WBE proposes FMR of the organism is the product of average BMR and number of cells [8]. This version not only attributes no role to the efficiency of the organization of the cells of W, but also clashes with the fact that, at under 25% ME, BMR exceeds FMR for all W. Ws of this sort are seen in such creatures as parasitic worms and fungi embedded in a food source, or small mammals that routinely function at an average ME less than 25%. In fact the appearance of large mammals awaited the development of nervous systems that allowed for greater Ep, the rate at which coulombs are captured as neuronal ATP, for example, in the redox coupling that is neurogastric. This allowed average ME to exceed 25%. Otherwise more massive mammals at less than 25% ME would have lower FMRs and shorter lives. This phenomenon can be seen in dogs and cats. Larger dogs live shorter lives, with an average ME just below 25%, while larger cats, including tigers and mountain lions, live longer than dogs, and have an average ME just over 25%.

Ws that function at an ME less than 25% are generally able to benefit from the effects of caloric restriction (Es) on longevity. There is a slight cushion before FMR exceeds BMR, when ME goes over 25%. This cushion is missing for all organisms that function at or over 25% average ME. For all these latter organisms FMR exceeds BMR, and so the maximum potential life span of the creature is greater than that of its cells. It is important to note that average ME for the organism is the same for all its constituent parts. The organism determines ME by the success of its ability to capture energy from food or photosynthesis. It is shared ME that defines organism wholeness, and is the basis of biological organization. That is why there are stem cells for all somatic structures, including nervous tissue, for Ws whose average ME is greater than 25%; and why the effects of caloric restriction on longevity for these creatures is threatening to BMR, which drops dramatically as ME increases over 25%. Herein lies the secret to aging and its associated decrepitude. It is a matter of the antagonism between FMR and BMR. The same dynamic is characteristic of flora, where BMR for the cells, renewed with the seasons, allows the organism comprised of those cells to live longer than any of them.

The phenomenon known as *quorum sensing*, when bacterial cells start doing things as a colony that no bacterium has the energy to do on its own, is something that occurs when Es drives ME over 25%. At this time the FMRs for W exceed the BMRs of W's constituents, and further increases in mass of W increase its FMR. The bacterial colony is W. An example of quorum sensing is the creation of biofilms, whether in the gut or on coral, where bacterial films act as a net to ensnare tide-borne particles that may be subjected to Id/catabolic breakdown. No individual bacterium has the MR to create these films, or to synthesize the pili that are associated with energy equilibration throughout the bacterial colony responsible for the film [9]. Quorum sensing is what was behind the oldest fossils of bacterial colonies on earth, the stromatolites found in the shallows off the coast of Australia. Stromatolites are the structures left after bacterial colonies have been encased in and inundated by water-borne sediments.

What separates flora from fauna and bacteria is the electrical properties of the relevant biomass. An ohmmeter will read a resistance from one point to another on any fauna, or in a bacterial colony; but not so with flora. Flora are not dielectric; their extracellular structures act as an insulator, except at exceedingly high voltages. Flora do not then equilibrate chemical energy extracellularly, and are not capable of motor behavior, which must be distinguished from phototropism. Motor behavior involves the equilibration of energy captured by biomass in the catabolic breakdown of a food/energy source, and should be distinguished from seemingly-directed, intracellular traffic of molecules driven by electrical fields. Motor behavior extends from a bacterial flagellum or the rotary-mechanism of the ATP-synthase complex, to the peristalsis of smooth muscle responding to local conditions, or to the skeletal articulation of striated muscle driven by nervous system discharge of neuronal ATP hydrolysis (Id).

Because the sophisticated version of Kleiber's Law is mathematical, deductive inferences can be made from it that can be tested. The forces and pressures it purports to model, said to be responsible for the origins, evolution, development and replication of all life, and claimed to define the parameters within which all genetic variation must be limited, are still at work. The equation models how Ia at the basal level, appearing as the digestion of food, causes perturbations in BMR of key structures of the gut. These perturbations are in turn equilibrated by Ep at the field level, appearing as phosphorylation in the neuron. Equilibration of MR perturbation from the introduction of this energy is not yet complete, and involves further Id at the field level, where Ep is replaced by Id, associated with nervous system trophism. Extrapolation from additional file 1 suggest that, if life span is directly related to MR or recharge rate, and if the ME of the organism applies to its cells, then the life span of an ATP molecule is very brief before its energy is lost as heat (if it is not used for something else), in organisms with a high average ME (over 25%). In organisms of increased encephalization, where average ME exceeds 25% because of Ep rather than Es, this Id breakdown of ATP acts as a sort of trickle-charger, providing Ia to all post-synaptic structures. This effect accounts for the warm-bloodedness of mammals, especially large mammals, with the heat as lost power in transmission, and higher ATP turnover rates due to lower MRs for ATP-organic molecules consonant with higher organismic MEs.

This sort of trickle-charger transmission drives the motor activity necessary to acquire more food, and also occurs as nervous system trophism. The redox dynamic is Ia from food » Ep (field level neurogastric/redox coupling resulting in ATP synthesis) » Id (neuronal dephosphorylation) » Ia (basal level nervous system trophism). Ep from food sources occurring as mass not readily reversible through Id (e.g., fat instead of ATP, where Ia from food is converted to Ep at the basal level without neurogastric coupling at the field level), if pronounced, can result in mass increase interfering in the transmission necessary for motor activity and trophism. This is the point of empirical entry into testing a hypothetical inference from the math on a human W in a clinical setting. A battery discharging into the body at the electrochemical, peripheral nerve endings, the hypothesis suggests, will cause BMR for the cells of the post-synaptic structure to fluctuate in what is a simulation of the conversion of neuronal Id into somatic Ia (nervous system trophism). This simulated trophism acts on the secondary bio-battery aspect to induce anabolism there, by reversing key chemical reactions associated with metabolic functioning, like the hydrolysis of ATP or the catabolic breakdown of NADH to NAD^+^. The reversibility process has roots in the secondary-cell aspects of biomass, but without diminution of FMR from neuronal Id to power the reversibility. Effectively, BMR and FMR are increased simultaneously, without the need for neuronal Id to complete energy distribution from gastric to somatic cells and structures. If effective ME at the basal level is reduced to less than 25%, energy will be absorbed as increased mass, occurring as increased protein density. When the somatic structure is muscle, muscle cell mass will be seen to increase. Ep and Ia will be triggered simultaneously, without antagonism between the two, without increased consumption of food, and without antagonism between BMR and FMR. The discharging battery acts as a second stomach, with its anode acting as nervous system neuronal Id. Over time mass increase will enhance systemic efficiency by acting upon the density of the protein structure of the organism's cells, such that average ME and FMR increase, extending life span of Ws over one gram in size, without the normal drops in BMR associated with increased ME in organisms of that size.

There are 0.86 nutritional calories in 1 watt-hour. If an open circuit voltage of 100 volts DC pulse-delivers an average of 30 milliamperes at a frequency of 900 Hz. for one hour, the watt-sec rating is 0.168, and that translates to 604 watt-hours. Delivered transcutaneously to the body's 1,152 motor endplate region/neuromuscular junctions for 1 second each, the result is 0.32 hours of stimulation, for 193.3 watt-hours. This means 166.2 calories were introduced to biomass W from the battery. The question then becomes, how well were the watts absorbed so that organism Ep and basal Ia are affected, and FMR and BMR are increased with this momentary change in ME?

A 75 kg human with an ME of 31% will have a maximum-potential-life-span (MPLS)/FMR of 8.8. A caloric intake of 2000 calories/day translates to 620 calories converted to ATP, motor behavior and protein mass, while the rest is lost as heat, or passed as undigested matter. If electrochemical stimulation increases ATP and protein synthesis at 100% efficiency, effective calories from food increases from 620 to 786. This puts ME at 39% momentarily, and raises MPLS to well over 200 years (if an MPLS of 8.8 is taken to be roughly equivalent to 88 years) – if that ME is sustained as an average. But it is not. It is momentary, though it does have an effect on average ME.

Capture of energy, as ATP/protein synthesis from electrochemical stimulation, is limited to the same restrictions that govern the turning of Id to Ia through exercise. The effective conversion of energy from Id to Ia is limited by the resistance to transmission of energy from the chemical synapse at the nerve ending to the electrical synapses at the gap junctions of the cells of somatic structures where ion channels form electrochemical salt bridges to the intracellular space. Transmission on smooth and striated muscle is dependent upon the cross-sectional area of the Type II muscle fiber. Muscle mass is determined by this cross-sectional area. Weak muscles have diminished cross-sectional area, and are hard to build and restore, regardless of how much activity or exercise is involved, because the power delivered to them is dependent upon their ability to absorb it, and this ability is dependent upon their fitness. The less the cross-sectional area, the less likely it is that Id can drive down Ia to less than 25%, a requirement for the absorption of energy as muscle mass. Prolonged cutaneous exposure to the anode can overwhelm the skin cells with an Ia they can't absorb, causing them to blister and burst as Ia is converted to Id for these cells. Skin irritation from electrical stimulation of muscle traditionally has been avoided through the use of a biphasic voltage waveform. This type of waveform prevents the use of electrochemistry, only possible with a monophasic waveform. This is why electrical muscle stimulators have not been able traditionally to build muscle. Biphasic waveforms cannot increase Type II fiber cross-sectional area necessary for muscle-mass building because they do not pass amperage.

## Conclusions: testing the equation

If, over time, average ME of the 75 kg human biomass is increased by 8%, going from 31% ME to 33.5%, from changes in the histology of the biomass, MPLS increases from 8.8 to 17.3, more than doubling possible life span. If average ME is increased only to 31.5%, MPLS/FMR increases to only 10.0. These are deductions from the equation. If the inferred predictions are found, upon testing, to increase human life span, and to build muscle mass, this will offer support to the idea that sophisticated Kleiber does indeed truly model the forces and pressures that are behind the appearance of life from chemistry, without resort to the particularities of cause and effect.

Other testable predictions present themselves. The equation suggests that, given the wide range of flatworm genome mass, average genome mass for parasitic flukes will be far less than that for planarians, on the basis of an ME reflecting the eating habits and lifestyle of the two types of flatworm. The equation suggests that birds live longer than rats of equal mass because the birds, eating less, operate at a higher ME than rats – with rats at or around 20%, and birds at or around 30%. This is also why bigger birds live longer lives, while bigger rats live shorter lives. The equation implies that the Id that leads to genetic mutation is more likely to occur in creatures that operate at higher ME, where either food supply is barely adequate, or where neurogastric energy distribution, from Id to Ia at the basal level, is impeded by histological deterioration, as in the aged and infirm. In the former case what is seen is species diversification, while in the latter what is seen is cancer and degeneration. Inferences from the math, including the application of electrochemistry to ameliorate or mitigate problems of health and fitness, need to be addressed.

## Competing interests

The author declares that he has no competing interests.

## Supplementary Material

Additional file 1 **Metabolic rates for a range of biomass vs. metabolic efficiency**. the data provided present metabolic rate/lifespan in numbers, with metabolic efficiency increasing from top to bottom, and biomass in grams from left to right. Highlighted values are those that appear on the graph in Figure 1. Notice the seams at which the value for metabolic rate remains constant. They occur at one gram and 25% efficiency, at which the value for metabolic rate is 1.0. In terms of lifespan, when compared to observed-life-spans of organisms big and small, the value of 1.0 approximates around ten years. Click here for file
