# Supplementary material for: The terrestrial evolution of metabolism and life – by the numbers
Source: Theor Biol Med Model. 2009 Aug 27;6:17. doi: 10.1186/1742-4682-6-17 (PMC2751747; doi:10.1186/1742-4682-6-17)
Supplement: Additional file 1 — Metabolic rates for a range of biomass vs. metabolic efficiency. the data provided present metabolic rate/lifespan in numbers, with metabolic efficiency increasing from top to bottom, and biomass in grams from left to right. Highlighted values are those that appear on the graph in Figure 1. Notice the seams at which the value for metabolic rate remains constant. They occur at one gram and 25% efficiency, at which the value for metabolic rate is 1.0. In terms of lifespan, when compared to observed-life-spans of organisms big and small, the value of 1.0 approximates around ten years. [file 1742-4682-6-17-S1.pdf]

	Mass in grams		1µg			1mg			1g			1kg			1,000kg
Eff.	1.E-09	1.E-07	1.E-06	1.E-05	1.E-04	1.E-03	1.E-02	1.E-01	1.E+00	1.E+01	1.E+02	1.E+03	1.E+04	1.E+05	1.E+06
1%	#######	#######	#######	#######	#######	#######	#######	#######	1.0000	0.00000	0.00000	0.00000	0.00000	0.00000	0.00000
5%	#######	#######	#######	#######	#######	#######	100000000	10000	1.0000	0.00010	0.00000	0.00000	0.00000	0.00000	0.00000
6%	#######	#######	#######	#######	#######	#######	2154435	1468	1.0000	0.00068	0.00000	0.00000	0.00000	0.00000	0.00000
7%	#######	#######	#######	#######	#######	51794747	138950	372.8	1.0000	0.00268	0.00001	0.00000	0.00000	0.00000	0.00000
8%	#######	#######	#######	#######	316227766	2371374	17783	133.4	1.0000	0.00750	0.00006	0.00000	0.00000	0.00000	0.00000
9%	#######	#######	#######	774263683	12915497	215443	3594	59.95	1.0000	0.01668	0.00028	0.00000	0.00000	0.00000	0.00000
10%	#######	#######	#######	31622777	1000000	31623	1000	31.62	1.0000	0.0316	0.00100	0.00003	0.00000	0.00000	0.00000
11%	#######	811130831	43287613	2310130	123285	6579	351.1	18.74	1.0000	0.0534	0.0028	0.00015	0.00001	0.00000	0.00000
12%	#######	38311868	3162278	261016	21544	1778	146.8	12.12	1.0000	0.0825	0.0068	0.00056	0.00005	0.00000	0.00000
13%	203091762	2894266	345511	41246	4924	587.8	70.17	8.377	1.0000	0.1194	0.0143	0.00170	0.00020	0.00002	0.00000
14%	11787686	316228	51795	8483	1389	227.6	37.28	6.105	1.0000	0.1638	0.0268	0.00439	0.00072	0.00012	0.00002
15%	1000000	46416	10000	2154.4	464.2	100.0	21.54	4.642	1.0000	0.2154	0.0464	0.01000	0.00215	0.00046	0.00010
16%	115478	8660	2371	649.4	177.8	48.70	13.34	3.652	1.0000	0.2738	0.0750	0.02054	0.00562	0.00154	0.00042
17%	17191	1968	666	225.4	76.27	25.81	8.733	2.955	1.0000	0.3384	0.1145	0.03875	0.01311	0.00444	0.00150
18%	3162	527.5	215.4	87.99	35.94	14.68	5.995	2.448	1.0000	0.4084	0.1668	0.06813	0.02783	0.01136	0.00464
19%	695.2	162.4	78.48	37.93	18.33	8.859	4.281	2.069	1.0000	0.4833	0.2336	0.11288	0.05456	0.02637	0.01274
20%	177.8	56.23	31.62	17.78	10.000	5.623	3.162	1.778	1.0000	0.5623	0.3162	0.1778	0.1000	0.0562	0.0316
21%	51.79	21.54	13.89	8.962	5.780	3.728	2.404	1.551	1.0000	0.6449	0.4160	0.2683	0.1730	0.1116	0.0720
22%	16.88	9.006	6.579	4.806	3.511	2.565	1.874	1.369	1.0000	0.7305	0.5337	0.3899	0.2848	0.2081	0.1520
23%	6.062	4.062	3.325	2.721	2.228	1.823	1.492	1.222	1.0000	0.8185	0.6700	0.5484	0.4489	0.3675	0.3008
24%	2.371	1.957	1.778	1.616	1.468	1.334	1.212	1.101	1.0000	0.9085	0.8254	0.7499	0.6813	0.6190	0.5623
25%	1.0000	1.0000	1.0000	1.0000	1.0000	1.0000	1.0000	1.0000	1.0000	1.0000	1.0000	1.0000	1.0000	1.0000	1.0000
26%	0.4507	0.5380	0.5878	0.6422	0.7017	0.7667	0.8377	0.9152	1.0000	1.093	1.194	1.304	1.425	1.557	1.701
27%	0.2154	0.3030	0.3594	0.4262	0.5055	0.5995	0.7110	0.8432	1.0000	1.186	1.407	1.668	1.978	2.346	2.783
28%	0.1086	0.1778	0.2276	0.2913	0.3728	0.4771	0.6105	0.7814	1.0000	1.280	1.638	2.096	2.683	3.433	4.394
29%	0.0574	0.1083	0.1487	0.2043	0.2807	0.3857	0.5298	0.7279	1.0000	1.374	1.887	2.593	3.562	4.894	6.723
30%	0.0316	0.0681	0.1000	0.1468	0.2154	0.3162	0.4642	0.6813	1.0000	1.468	2.154	3.162	4.642	6.813	10.00
31%	0.0181	0.0442	0.0690	0.1077	0.1682	0.2626	0.4101	0.6404	1.0000	1.562	2.438	3.808	5.946	9.284	14.50
32%	0.0107	0.0294	0.0487	0.0806	0.1334	0.2207	0.3652	0.6043	1.0000	1.655	2.738	4.532	7.499	12.41	20.54
33%	0.0066	0.0201	0.0351	0.0614	0.1072	0.1874	0.3275	0.5722	1.0000	1.748	3.054	5.337	9.326	16.30	28.48
34%	0.0041	0.0140	0.0258	0.0475	0.0873	0.1607	0.2955	0.5436	1.0000	1.840	3.384	6.225	11.45	21.06	38.75
35%	0.0027	0.0100	0.0193	0.0373	0.0720	0.1389	0.2683	0.5179	1.0000	1.931	3.728	7.197	13.89	26.83	51.79
36%	0.0018	0.0073	0.0147	0.0297	0.0599	0.1212	0.2448	0.4948	1.0000	2.021	4.084	8.254	16.68	33.71	68.13
37%	0.0012	0.0054	0.0113	0.0239	0.0504	0.1064	0.2246	0.4739	1.0000	2.110	4.453	9.397	19.83	41.84	88.30
38%	0.0008	0.0040	0.0089	0.0195	0.0428	0.0941	0.2069	0.4549	1.0000	2.198	4.833	10.62	23.36	51.35	112.9
39%	0.0006	0.0031	0.0070	0.0160	0.0367	0.0838	0.1914	0.4375	1.0000	2.285	5.223	11.94	27.28	62.36	142.5
40%	0.0004	0.0024	0.0056	0.0133	0.0316	0.0750	0.1778	0.4217	1.0000	2.371	5.623	13.34	31.62	74.99	177.8
41%	0.0003	0.0019	0.0046	0.0112	0.0275	0.0675	0.1658	0.4072	1.0000	2.456	6.032	14.82	36.39	89.38	219.5
42%	0.0002	0.0015	0.0037	0.0095	0.0240	0.0611	0.1551	0.3938	1.0000	2.540	6.449	16.38	41.60	105.6	268.3
43%	0.0002	0.0012	0.0031	0.0081	0.0212	0.0555	0.1455	0.3814	1.0000	2.622	6.874	18.02	47.25	123.9	324.8
44%	0.0001	0.0009	0.0026	0.0069	0.0187	0.0506	0.1369	0.3700	1.0000	2.703	7.305	19.74	53.37	144.2	389.9
45%	0.0001	0.0008	0.0022	0.0060	0.0167	0.0464	0.1292	0.3594	1.0000	2.783	7.743	21.54	59.95	166.8	464.2
50%	0.00003	0.00032	0.00100	0.00316	0.01000	0.03162	0.10000	0.3162	1.0000	3.162	10.000	31.62	100.0	316.2	1000
55%	0.00001	0.00015	0.00053	0.00187	0.00658	0.02310	0.08111	0.2848	1.0000	3.511	12.33	43.29	152.0	533.7	1874
60%	0.00001	0.00008	0.00032	0.00121	0.00464	0.01778	0.06813	0.2610	1.0000	3.831	14.68	56.23	215.4	825.4	3162
65%	0.00000	0.00005	0.00020	0.00084	0.00346	0.01425	0.05878	0.2424	1.0000	4.125	17.01	70.17	289.4	1194	4924
70%	0.00000	0.00003	0.00014	0.00061	0.00268	0.01179	0.05179	0.2276	1.0000	4.394	19.31	84.83	372.8	1638	7197
75%	0.00000	0.00002	0.00010	0.00046	0.00215	0.01000	0.04642	0.2154	1.0000	4.642	21.54	100.0	464.2	2154	10000
80%	0.00000	0.00002	0.00007	0.00037	0.00178	0.00866	0.04217	0.2054	1.0000	4.870	23.71	115.5	562.3	2738	13335
85%	0.00000	0.00001	0.00006	0.00030	0.00150	0.00763	0.03875	0.1968	1.0000	5.080	25.81	131.1	666.1	3384	17191
90%	0.00000	0.00001	0.00005	0.00024	0.00129	0.00681	0.03594	0.1896	1.0000	5.275	27.83	146.8	774.3	4084	21544
95%	0.00000	0.00001	0.00004	0.00021	0.00113	0.00616	0.03360	0.1833	1.0000	5.456	29.76	162.4	885.9	4833	26367
100%	0.00000	0.00001	0.00003	0.00018	0.00100	0.00562	0.03162	0.1778	1.0000	5.623	31.62	177.8	1000	5623	31623
